# Awareness of Diabetic Patients in the Qassim Region About Diabetic Foot and Its Complications

**DOI:** 10.7759/cureus.52306

**Published:** 2024-01-15

**Authors:** Ahmed Alotaibi, Nawaf Alqhtani, Abdulaziz Alluhaymid, Lulwah Alhomaidan, Meshal Alwabel, Abdulaziz Algurafi, Yassir Alenizi, Omer A AsSaigal

**Affiliations:** 1 Surgery, Unaizah College of Medicine and Medical Sciences, Qassim University, Buraydah, SAU; 2 General Surgery, Qassim University, Buraydah, SAU; 3 College of Medicine, Unaizah College of Medicine and Medical Sciences, Qassim University, Buraydah, SAU; 4 Endocrinology and Diabetes, Diabetes and Endocrine Center, Buraydah, SAU; 5 Diabetes and Endocrinology, King Saud Hospital, Unaizah, SAU

**Keywords:** foot protoction, complications, awareness, diabetic foot ulcer, diabetes

## Abstract

Introduction

Diabetes mellitus (DM) is a chronic condition brought on by either insufficient insulin production by the pancreas or inefficient insulin utilization by the body. A hormone called insulin controls blood sugar. Patients with type 1 or type 2 diabetes frequently experience diabetes complications, which are also a major cause of morbidity and mortality. Microvascular and macrovascular problems of diabetes are the two main categories, with the former having a significantly higher prevalence than the latter. In contrast to macrovascular problems, which include cardiovascular disease, stroke, and peripheral artery disease (PAD), microvascular sequelae include neuropathy, nephropathy, and retinopathy. The occurrence of a foot ulcer coupled with neuropathy, PAD, and infection is known as diabetic foot (DF) syndrome, and it is a primary factor in lower limb amputation. Finally, there are additional diabetes problems that fall outside of the two categories listed before, including birth defects, dental disease, and decreased infection resistance.

Aim

This study aimed to evaluate the awareness of diabetic patients in the Qassim region about diabetic foot and its complications.

Patient and methods

This retrospective cohort study was conducted between January 2021 and January 2022 among diabetic patients. The patients were contacted through the contact numbers listed in their medical charts at the Diabetic Center in King Saud Hospital in Unaizah and the Diabetes Center in King Fahad Specialist Hospital. The data were collected by sending the link to the targeted patients using the Google Form questionnaire.

Results

Of the 384 diabetic patients, 51.6% were females, and 28.6% were aged between 18 and 30 years old. A previous history of foot ulcers has been reported by 10.4%. The overall mean score was 11.3 (SD 2.99) out of 20 points, with poor, moderate, and good awareness levels constituting 25.8%, 66.4%, and 7.8%, respectively. Factors associated with increased awareness include younger age, female gender, having no associated chronic disease, and not experiencing soreness on the foot or leg.

Conclusion

There was modest awareness among the diabetic population regarding diabetes foot care and its complications. Independent significant predictors of increased knowledge include younger age, female gender, having no associated chronic disease, and not experiencing soreness on the foot or leg. Increased diabetic education is vital to improving awareness levels of diabetic foot complications.

## Introduction

Diabetes mellitus (DM) is a chronic condition brought on by either insufficient insulin production by the pancreas or inefficient insulin utilization by the body. A hormone called insulin controls blood sugar [1]. Patients with type 1 or type 2 diabetes frequently experience diabetes complications, which are also a major cause of morbidity and mortality. Microvascular and macrovascular problems of diabetes are the two main categories, with the former having a significantly higher prevalence than the latter [2]. In contrast to macrovascular problems, which include cardiovascular disease, stroke, and peripheral artery disease (PAD), microvascular sequelae include neuropathy, nephropathy, and retinopathy. The occurrence of a foot ulcer coupled with neuropathy, PAD, and infection is known as diabetic foot (DF) syndrome, and it is a primary factor in lower limb amputation [3]. Finally, there are additional diabetes problems that fall outside of the two categories listed before, including birth defects, dental disease, and decreased infection resistance [3]. The number of DM patients in Saudi Arabia (SA), which is the second-richest country in the Arab world and seventh-richest overall, is on the rise. The prevalence of diabetes mellitus (DM), primarily type 2 diabetes (T2DM), is estimated to be around 7 million people in Saudi Arabia [4]. A recent study reported the incidence of DM to be around 23.4% in the Saudi population, with a higher incidence in men than in women [5]. Additionally, SA has been ranked among high-risk nations regarding overweight and obesity, which is a major challenge for the country's public health [6]. There is a strong correlation between obesity, DM, hypertension, and over 20,000 deaths each year in SA, where children and teenagers have a high prevalence of obesity [6,7].

## Materials and methods

Study design and study setting

A retrospective cohort study was conducted in the period between January 2021 and January 2022. Medical records of diabetic patients attending the diabetic center at King Saud Hospital in Unaizah and the endocrine and diabetes center at King Fahad Specialist Hospital, Qassim region, were our target.

Sample size

The sample size has been calculated using Epi-Info software with a confidence interval (CI) of 95%, a power of 80%, and an expected level of awareness of 50%. The calculated sample size is 380.

Sampling technique**

A convenient non-probability sampling technique was used. The contact numbers of the diabetic patients attending the diabetic center at King Saud Hospital in Unaizah and the endocrine and diabetes center at King Fahad Specialist Hospital were obtained from the medical records of diabetic patients. 

Inclusion criteria**

Diabetic patients attending the selected diabetic center, aged 18 years or older, of both sexes and any nationality, can read and write, have a social media account, and are accepted to participate in the study.

Exclusion criteria 

Diabetic patients from outside the selected diabetic center can’t read or write, have no social media accounts, and refuse to participate in the study.

Data collection methods**

In this study, we applied a predesigned questionnaire prepared by the authors based on a valid and reliable scale to measure diabetic patients’ awareness of diabetic foot and its complications. A self-administered, structured online survey on a Google Form was sent to all diabetic patients enlisted in the medical records of the selected diabetic center. The aim of the study was announced in the questionnaire. The questionnaire contained two sections: 1) Socio-demographic data includes (age in years, gender, residence, nationality, educational level, occupation, and chronic illnesses). 2) Diabetes Care Program of Nova Scotia (DCPNS) questionnaire [8]. 

The DCPNS questionnaire has 30 items: items from 1-3 to evaluate the history of foot problems, items from 4-8 to evaluate current foot problems, items from 9-14 to evaluate foot care, items from 15-16 to evaluate footwear, items from 17-26 to evaluate safety and prevention, and items from 27-30 to evaluate foot care education. 

Yes and no; other questions with specific answers are the response options. Each correct response received a score of one, for a total of 30 points. A score of less than 50% (less than 15 points) means having low knowledge about diabetic foot; a correct score of 50%-69% (15-21 points) means fair understanding; and a score of more than 70% (more than 21 points) means having good knowledge about diabetic foot. The participants were subdivided into two groups: those with inadequate knowledge and those with equitable knowledge.

The DCPNS questionnaire is both a valid and reliable scale that is often used to measure diabetic foot awareness. However, the questionnaire has to be translated into the Arabic language. We translated the questionnaire into an Arabic version and were assessed by two experts to evaluate its reliability after translation. Moreover, a pilot study was conducted with 30 participants. Their results were excluded from the study to test the clarity and understandability of the questionnaire and to calculate the time needed to complete the questionnaire. 

Questionnaire criteria

The awareness of diabetic patients toward diabetic foot and its complications has been assessed using a 20-item questionnaire with "yes" coded with 1 and "no" coded with 0 as answer options. Negative questions have been re-coded in reverse order to avoid bias in the score. The total awareness score has been calculated by adding all 20 items. A score ranging from 0 to 20 points has been generated. The greater the score, the greater the awareness of diabetic foot and its complications. By using 50% and 75% as cutoff points to determine the level of awareness. Patients were considered to have poor awareness if the score was below 50%; 50% to 75% were considered moderate; and levels above 75% were considered good awareness.

Statistical analysis

All categorical data were given as frequencies and proportions (%). All continuous data were calculated and summarized as mean and standard deviations. The awareness score was compared with the socio-demographic characteristics and the prevalence of diabetic foot by using the Mann-Whitney Z-test. The normality test (i.e., statistical collinearity) was performed using the Shapiro-Wilk test as well as the Kolmogorov-Sminov test. The total awareness score follows a non-normal distribution. Therefore, the non-parametric test was applied. Statistical significance was established at the p<0.05 level and highly statistical significance at the p<0.001 level. All statistical data were performed and analyzed using IBM Corp. Released 2019. IBM SPSS Statistics for Windows, Version 26.0. Armonk, NY: IBM Corp.

Ethical considerations

The aim of the study was explained to all participants before conducting the questionnaire. Both the confidentiality and privacy of the information given by participants were firmly secured. The ethical approval was obtained from the Regional Ethical Committee.

## Results

This study enrolled 384 diabetic patients. As described in Table 1, 110 (28.6%) were aged between 18 and 30 years old, with more than half (198 [51.6%]) being females. (197 [51.3%]) lived in Buraidah, and nearly half (190 [49.5%]) were bachelor's degree holders. Unemployed patients constituted 220 (57.3%). The most commonly associated chronic disease was cholesterol (153 [39.8%]). In addition, 221 (57.6%) had a duration of diabetes of more than 10 years.

**Table 1:** Socio-demographic characteristics of the patient with diabetes (n=384) † Some patients have more than one chronic disease.

Study variables	N (%)
Age group	
18 to 30 years	110 (28.6%)
31 to 40 years	67 (17.4%)
41 to 50 years	68 (17.7%)
51 to 60 years	78 (20.3%)
>60 years	61 (15.9%)
Gender	
Male	186 (48.4%)
Female	198 (51.6%)
Residence city	
Buraidah	197 (51.3%)
Unaizah	129 (33.6%)
Others	58 (15.1%)
Educational level	
Uneducated	25 (06.5%)
High school or below	152 (39.6%)
Bachelor's degree	190 (49.5%)
Postgraduate	17 (04.4%)
Employment status	
Unemployed	220 (57.3%)
Health sector	19 (04.9%)
Non-health sector	77 (20.1%)
Non-office work	29 (07.6%)
Office work	39 (10.2%)
Associated chronic disease ^†^	
None	176 (45.8%)
Cholesterol	153 (39.8%)
Hypertension	99 (25.8%)
Other	52 (13.5%)
Diabetes duration	
1 to 5 years	93 (24.2%)
6 to 10 years	70 (18.2%)
>10 years	221 (57.6%)

In Table 2, 84 (21.9%) of the patients reported having a sore or cut on the foot or leg that took two weeks to heal. The prevalence of patients who had a previous history of foot ulcers was 10.4% (n=40). There were five patients (1.3%) who got their toe, foot, or leg amputated. Forty-one (10.7%) had concurrent ulcers, soreness, or blisters on their feet. Four cases (1%) had blood or discharge on their socks. The prevalence of patients who have calluses on their feet was 14.1% (n=54). Also, 197 (51.3%) experienced numbness, tingling, pins and needles, or an itching sensation on their feet. Additionally, 154 (40.1%) experienced tightness, heaviness, pain, or cramps in their feet or legs.

**Table 2:** Assessment of patients’ awareness about diabetic foot and its complications (n=384)

Statement	N (%)
Do you use medicated products for warts, corns, or calluses? [no]	370 (96.4%)
Do you wash your feet every day? [yes]	359 (93.5%)
Do you smoke? [no]	332 (86.5%)
Can you reach and see the bottoms of your feet? [yes]	319 (83.1%)
Do you cut your own toenails? [yes]	317 (82.6%)
Would you like a handout on how to care for your feet? [yes]	286 (74.5%)
Do you sit with your legs crossed? [yes]	267 (69.5%)
Do you always inspect your shoes for foreign objects or torn linings? [yes]	258 (67.2%)
Do you examine your feet? [yes]	255 (66.4%)
Do you always test the water temperature before putting your foot in? [yes]	241 (62.8%)
Do you use a moisturizing cream on your feet? [yes]	219 (57.0%)
Do you dry well between the toes? [yes]	195 (50.8%)
Do you ever soak your feet? [yes]	175 (45.6%)
Have you ever read any handouts on foot care? [yes]	160 (41.7%)
Do you put moisturizing cream or lotions between your toes? [yes]	144 (37.5%)
Have you ever read any handouts on proper footwear? [yes]	136 (35.4%)
Do you ever wear shoes without wearing socks? [no]	107 (27.9%)
Do you ever walk around in your bare feet? [no]	105 (27.3%)
Have you ever attended a class on how to care for your feet? [yes]	40 (10.4%)
Do you use a hot water bottle or heating pad on your feet? [yes]	38 (09.9%)
Total awareness score (mean ± SD)	11.3 ± 2.99
Level of awareness	
Poor	99 (25.8%)
Moderate	255 (66.4%)
Good	30 (07.8%)

Regarding the assessment of patients' awareness of diabetic foot and its complications (Table 3), the most notable statements where the diabetic patients showed higher awareness include "Not to use medicated products for warts, corns, or calluses" (370 [96.4%]), "Washing of feet every day" (359 [93.5%]), "Not smoking" (332 [86.5%]), "Reaching and seeing the bottoms of feet (319 [83.1%]), and "Cutting own toenails" (317 [82.6%]). The overall mean awareness score was 11.3 (SD 2.99). Accordingly, 255 (66.4%), 99 (25.8%), and 30 (7.8%) were considered to have moderate, poor, and good awareness levels. 

**Table 3:** Prevalence of diabetic foot ulcers among the diabetic population (n=384)

Statement	N (%)
Have you ever had a sore or cut on your foot or leg that took more than two weeks to heal?	
No	300 (78.1%)
Yes	84 (21.9%)
Have you ever had a foot ulcer?	
No	344 (89.6%)
Yes	40 (10.4%)
Have you ever had an amputation of a toe, foot, or leg?	
No	379 (98.7%)
Yes	05 (01.3%)
Do you have an ulcer, sore, or blister on your feet at this time?	
No	343 (89.3%)
Yes	41 (10.7%)
Do you have blood or discharge on your socks?	
No	380 (99.0%)
Yes	04 (01.0%)
Do you have any calluses on your feet?	
No	330 (85.9%)
Yes	54 (14.1%)
Do you have numbness, tingling, pins and needles, or itching sensation in your feet?	
No	187 (48.7%)
Yes	197 (51.3%)
Do you have any tightness, heaviness, pain, or cramps in your feet or legs?	
No	230 (59.9%)
Yes	154 (40.1%)

**Figure 1:**
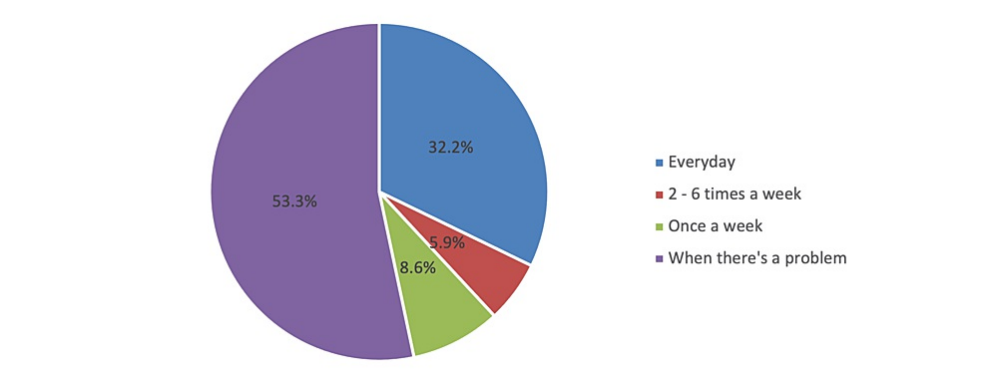
Frequency of examining one’s own feet

In Figure 1, among patients who examined their feet (n=255), 136 (53.3%) examined their feet when there was a problem, and 82 (32.2%) examined them every day. 

**Figure 2:**
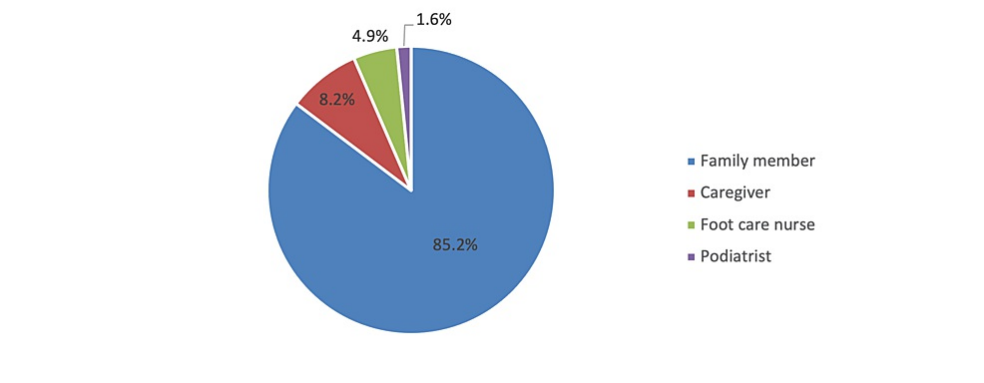
Persons who provided help when cutting toenails

In Figure 2, among patients who provided care when cutting nails (n=61), the most common individual who provided help was a family member (52 [85.2%]).

**Figure 3:**
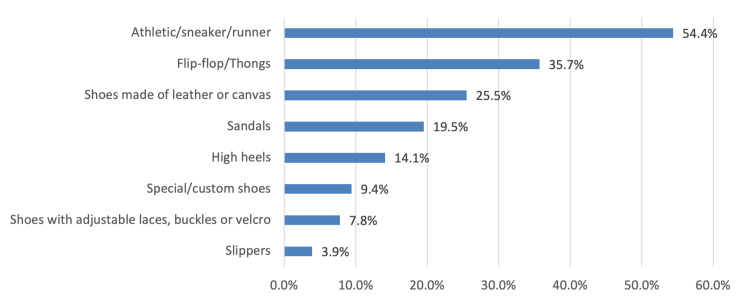
Types of shoes patients used to wear

In Figure 3, the most common shoes that the patients used to wear were athletic/sneaker/runner (209 [54.4%]), followed by flip-flops/thongs (137 [35.7%]) and shoes made of leather or canvas (98 [25.5%]).

**Figure 4:**
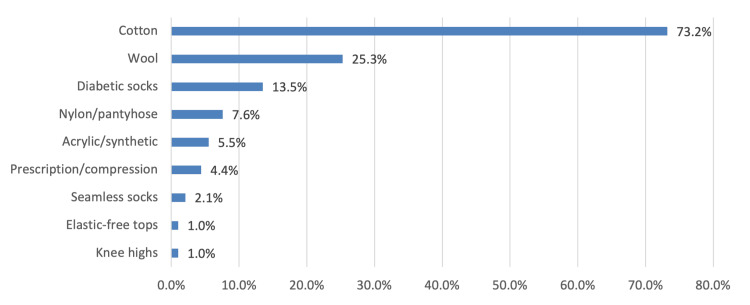
The kind of socks patients used to wear

In Figure 4, the most common socks that patients used to wear were cotton (281 [73.2%]), followed by wool (97 [25.3%]), and diabetic socks (52 [13.5%]).

When measuring the association between the scores of awareness according to the socio-demographic characteristics and the frequency of diabetic foot ulcers (Table 4), it was observed that a higher awareness score was more associated with a younger age group (Z=2.122; p=0.034), gender female (Z=6.200; p<0.001), those without associated chronic disease (Z=2.268; p=0.023) and having no sore or cut on foot or leg that took more than two weeks to heal (Z=2.587; p=0.010). 

**Table 4:** Differences in the score of awareness in relation to socio-demographic characteristics and the prevalence of diabetic foot ulcers (n=384) § P-value has been calculated using the Mann-Whitney Z-test. ** Significant at the p<0.05 level. *** Highly significant at the p<0.001 level.

Factor	Awareness Score (20) Mean ± SD	Z-test	P-value ^§^
Age group			
≤40 years	11.6 ± 2.92	2.122	0.034 **
>40 years	10.9 ± 3.04		
Gender			
Male	10.3 ± 2.76	6.200	<0.001 ***
Female	12.1 ± 2.96		
Educational level			
High school or below	10.9 ± 3.19	1.648	0.099
Bachelor or higher	11.5 ± 2.81		
Employment status			
Unemployed	11.3 ± 3.04	0.116	0.908
Employed	11.2 ± 2.95		
Associated chronic disease			
No	11.6 ± 2.84	2.268	0.023 **
Yes	10.9 ± 3.10		
Diabetes duration			
≤10 years	11.0 ± 2.79	1.767	0.077
>10 years	11.4 ± 3.14		
Have you ever had a sore or cut on your foot or leg that took more than two weeks to heal?			
No	11.5 ± 3.02	2.587	0.010 **
Yes	10.6 ± 2.83		
Have you ever had a foot ulcer?			
No	11.3 ± 2.99	0.779	0.436
Yes	10.9 ± 3.07		
Do you have any calluses on your feet?			
No	11.3 ± 3.01	0.883	0.377
Yes	10.9 ± 2.92		
Do you have numbness, tingling, pins and needles, or itching sensation in your feet?			
No	11.1 ± 3.08	1.044	0.296
Yes	11.4 ± 2.92		
Do you have any tightness, heaviness, pain or cramps in your feet or legs?			
No	11.2 ± 2.99	0.541	0.588
Yes	11.4 ± 3.02		

## Discussion

DM patients are prone to developing diabetic foot ulcers, which could lead to infection, amputation, gangrene, and eventually mortality due to multiorgan failure and sepsis [9,10]. Hence, a preemptive approach is necessary to avoid any diabetic foot complications by providing continuous education among patients and their caregivers. The present study is carried out to determine the level of awareness among diabetic patients toward diabetes and its complications. In this study, there was adequate awareness about diabetic foot complications among our population. Based on our criteria, 66.4% (N=255) were categorized as having moderate awareness, 7.8% (N=30) were good, and 25.8% (N=99) had poor awareness levels (mean score: 11.3; SD 2.99, out of 20 points). This is consistent with the study of Algshanen et al. (2017) [11]. Based on their reports, 55.1% of the respondents got high scores on at least seven to eight out of eight knowledge questions. This has been concurred by the paper of Shamim et al. (2021) [12], with a mean knowledge score of 8.58 out of 12 questions suggesting that the majority of the patients had sufficient knowledge about diabetes foot complications. Contradicting these reports, several studies have documented poor knowledge of diabetic foot ulcers and their complications [13-16]. The research done by Aldawish et al. (2018) [13] reported the lowest prevalence of poor knowledge, as 90% of the DM patients indicated having no previous education about diabetic foot disease (DFD). Diabetic patients need to understand DFD well to provide appropriate care for their feet. Hence, clinicians should exert more effort to educate DM patients about the complications of diabetes.

Data in our study suggest that the younger age group, female gender, without having associated chronic disease or a history of sores or cuts on feet or legs, were identified as the independent significant predictors of increased awareness. Consistent with our reports, in Iran [15], the female gender was also identified as one of the significant predictors of higher knowledge regarding the prevention and care of diabetic foot ulcers (DFU). Other significant socio-demographic factors being identified were longer DM duration, previous history of DFU, and having a lower limb amputated due to DFU. In Nepal [17], illiterate patients were more likely to exhibit poor knowledge about foot care practices. However, in our study, we did not find any significant associations between awareness score in terms of education level, employment status, duration of diabetes, previous history of a foot ulcer, the experience of numbness, tingling, pins, and needed or itching sensation of feet, and experience of tightness, heaviness, pain, or cramps in feet or legs (all p>0.05). This has been reflected in a study done in Uganda [16], where they found no significant association between knowledge about diabetic foot complications and all demographic variables (p>0.05). 

Regarding the specific assessment of awareness and practices of the diabetic foot and its complications, our results showed that, despite most of our patients being aware of some of its determinants, including not using medicated products for the treatment of skin diseases (370 [96.4%]), washing of feet every day (359 [93.5%]), avoidance of smoking (332 [86.5%]), practicing reaching the bottom of feet (319 [83.1%]), and self-cutting of toenails (317 [82.6%]), our population also showed poor awareness on several indicators of awareness. Most notably, about the use of a hot water bottle or heating pad on feet (38 [9.9%]), attendance to class on how to care for the feet (40 [10.4%]) and walking around with foot protection (105 [27.3%]). In a study conducted by Shamim et al. (2021) [12], even though a vast majority (73.7%) were cutting toenails regularly, only 38.4% were washing their feet on a daily basis, and the use of moisturizing cream was practiced by nearly one-third of the patients (30.4%). On the contrary, Shrestha et al. (2017) [17] documented that 68.9% of the DM patients did not practice washing their feet with warm water, with regularly walking barefoot being reported by 30.6%, while 26.5% were not frequently examining their footwear before wearing it. However, in our study, 66.4% were consistently examining their feet, although 53.3% only examined when there was a problem, which was consistent with the paper of Aldawish et al. (2018) [13].

Proper footwear and socks among diabetic patients are necessary to prevent diabetic foot complications. In our study, the most commonly preferred shoes were athletic/sneaker/runner shoes (209 [54.4%]), followed by flip-flops/thongs (137 [35.7%]) and shoes made of leather or canvas (98 [25.5%]), and only 15 (3.9%) preferred slippers. On the other hand, most of our patients preferred cotton socks (281 [73.2%]). Other types of socks were less preferred, including wool (97 [25.3%]), diabetic socks (52 [13.5%]), nylon/pantyhose (29 [7.6%]), acrylic/synthetic (21 [5.5%]), prescription/compression (17 [4.4%]), seamless socks (8 [2.1%]), elastic-free tops (4 [1%]), and knee highs (4 [1%]). Incidentally, Nancy et al. (2020) [18] reported that footwear with a hard shore value (footwear with a harder substance) was used by 42.9% of the patients. However, the author pointed out that the use of footwear with hard substances is hazardous for a diabetic neuropathic foot. Additionally, inadequate footwear awareness and practices were detected in 60% and 70.5% of the patients, respectively.

The limitations were that the results cannot be generalized to Saudi Arabia, our sampling technique is convenient and non-probability, and the data was a self-administered survey.

## Conclusions

Despite satisfactory awareness among diabetic patients toward diabetic foot and its complications, there were gaps that needed to be addressed. Younger female patients with no chronic disease and who did not suffer from long-term soreness in the foot or leg tended to be more aware of diabetic foot complications than the rest of the diabetic patients. This study underscores the need for more diabetic education to improve awareness of diabetic foot complications. This improvement can be accomplished through awareness programs designed for the prevention and/or early diagnosis of diabetes foot complications among patients with diabetes.
